# Predictability in Contemporary Medicine

**DOI:** 10.3389/fmed.2021.510421

**Published:** 2021-06-16

**Authors:** Michele M. Ciulla

**Affiliations:** ^1^Laboratory of Clinical Informatics and Cardiovascular Imaging, University of Milan, Milan, Italy; ^2^Department of Clinical Sciences and Community Health, University of Milan, Milan, Italy; ^3^Cardiovascular Diseases Unit, Fondazione IRCCS Cà Granda Ospedale Maggiore Policlinico, Milan, Italy

**Keywords:** diseases, Koch's postulates, multifactorial, risk factors, COVID-19, susceptibility, mathematical-statistical models, guidelines

## Abstract

Medical practice is increasingly coming under the guidance of statistical-mathematical models that are, undoubtedly, valuable tools but are also only a partial representation of reality. Indeed, given that statistics may be more or less adequate, a model is still a subjective interpretation of the researcher and is also influenced by the historical context in which it operates. From this opinion, I will provide a short historical excursus that retraces the advent of probabilistic medicine as a long process that has a beginning that should be sought in the discovery of the complexity of disease. By supporting the belonging of this evolution to the scientific domain it is also acknowledged that the underlying model can be imperfect or fallible and, therefore, confutable as any product of science. Indeed, it seems non-trivial here to recover these concepts, especially today where clinical decisions are entrusted to practical guidelines, which are a hybrid product resulting from the aggregation of multiple perspectives, including the probabilistic approach, to disease. Finally, before the advent of precision medicine, by limiting the use of guidelines to the original consultative context, an *aged* approach is supported, namely, a relationship with the individual patient.

“*Medicine is the most humane of all sciences, it is practiced by humans for human health”*.

## Introduction

The aim of this paper is to present in a historical perspective the process that led to the affirmation of the *mathematical-statistical models* in medicine as a surrogate system for making clinical decisions in order to bring it back to its original theoretical and rebuttable domain. The intent is not to give a sterile criticism but to support the proper use of any statistical-mathematical interpretative model, that is, subjective, fallible, and, therefore, confutable as any product of science. Since any constructive criticism must be proactive, in this article I will therefore support the recovery of the relationship with the individual patient that cannot be reduced to a mathematical average. The discussion will be articulated in a few points: the first will outline the process that led to the discovery of the complexity of disease and the advent of probabilistic medicine; the second, starting from the definition of illness as an unfavorable interaction between genes and environment, will support the general unpredictability of the disease as the result of an excess of variables whose control is very unlikely; the third point will try to demystify statistical-mathematical models by bringing them back to the main watercourse of science that should be understood as a continuous process of knowledge made of successes and failures; in this regard, in the fourth point, mathematics at the bedside will be supported as a tool that, till now, is the most profitable one in developing interpretative models in medicine; and the fifth point will criticize the formal application of guidelines as a hybrid product resulting from the aggregation of multiple instances, often incompatible, by supporting their original consultative role.

## Discussion

### The Question of Identifying the Causes of Diseases

In clinical medicine, to identify the causes of injury and/or illness is essential for the progress of knowledge and to prevent the progress of disease and/or to develop appropriate care; unfortunately, the simplified causative approach, well-summarized in *Koch's Postulates*, is no longer appropriate (1). This is because the outcome of illness, either healing or death, is never predictable with certainty but is, still, a causal inference based on the *study of probability*. Formulated to identify the *pathogen* responsible for a specific disease, the postulates contributed to the spread of an etiological approach based on a mono-factorial cause-effect relationship that has been replaced, since the Framingham Heart Study (2), in favor of a *multifactorial* one based on the concept of *risk factors*. This model has also expanded in the area of infectious diseases as even the Black Death of the 14th century, caused by Yersinia pestis, has been claimed to be a multifactorial pandemic (3) by postulating a series of causative factors that are reported in Table 1A.

**Table 1:** Prevalence, causation, and complexity of diseases.

**A. Factors affecting the prevalence and severity of infectious diseases.**
• Climatic factors
• Social conditions
• Molecular variations in pathogens
• Dynamic of the vector agent
• Genetics of the host population
• Interactions with concurrent diseases
**B. Cause vs. causal association.**
→ Cause
• any factor that produces an effect
• in medicine, also reported as etiology, pathogenesis, mechanism.
→ Causal association
• any factor that reveals an increased frequency of association in exposed vs. non-exposed
• in medicine, a factor associated is a risk factor.
**C. Complexity of individual adaptation.**
There are many degrees of freedom:
• the initial condition is uncertain
• the sensitivity/susceptibility is individual
• the future exposure to the environment depends on space-time
• different systems are dynamically involved
• the results may be of opposite sign, unfavorable, neutral, or even favorable.

The very recent emergence of SARS-CoV-2 and the COVID-19 pandemic, while pointing out the dynamic of spreading-over-species of infectious diseases (4), has provided an unexpected opportunity to link the puzzle of variable clinical manifestations and outcomes with host genomic factors (5).

Thus, all diseases, including the infectious ones are, possibly, multifactorial with an inherent increase in the complexity of the model since a cluster of interacting causative factors are, usually, associated and none of them are sufficient (6). From such a perspective, the association between events affecting biological life is not simply deterministic but more elusive as it follows a *non-linear dynamic* (7) making their effects, or outcomes, almost unpredictable in the individual subject. Thus, leaving behind the mono-factorial approach and focusing on the multi-factorial one, the main problem is to determine the probabilities of an event, such as illness or death, or the success of an intervention/treatment, whose degrees of freedom are innumerable. In Table 1B the main differences between cause and causal association are listed. Finally, in a clinical setting, when observing two events-diseases apparently connected with each other, with a possible causal relationship, it must be considered that there are, also, *recurring events*, sometimes cyclical, whose consequentiality may be casual and/or influenced by the direction, forward or backward, of the observation. Thus it may be difficult to reconstruct the time-line (8) like in the “*which came first: the chicken or the egg?”* dilemma.

### Why It Is Rather Impossible to Predict the Future for the Individual Subject

At present, a disease is the result of an *unfavorable* interaction between genes and environment, thus we must shift our focus to the *pattern of interaction* that has to do more with *individual adaptation*. Indeed, changes in the environment are handled by the same strategy that drives development and evolution by using biological resources involved in tissue maintenance and repair of damage (9). This kind of *susceptibility* is linked to a genetic risk as the disease manifests itself in a certain environmental context making the interaction unfavorable, and this is only known afterwards. Thus, we are facing *uncertainty* in the initial condition, with *many degrees of freedom* affecting susceptibility and future exposures depending on space-time and whether the environmental context is neutral or, sometimes, even favorable (10). This also explains the issue of s*elective advantages*, in terms of probability of survival, with the emergence of different *phenotypes* from a single *genotype* that are the result of the *epigenetic* machinery as proposed by Waddington in 1957. Having a unique genotype, more or less fixed, and an epigenome to provide dynamic and flexible responses to environmental changes does not simplify the matter, but rather makes it more complex as the individual process of adaptation is tricky (Table 1C). We can, therefore, say that no one is healthy and everyone is sick since the “*boundary between health and disease is, at least, fuzzy as it moves according to the reciprocal interaction between phenotype and environment and each individual is a different phenotype”* (11).

### Science and the Ability to Prevent and Treat Diseases Through the Forecasts

Science can be viewed as a process, carried out by scientists, aimed at increasing the knowledge of the internal/external human environment, through the formulation of relevant questions and using appropriate methodology for getting answers. The scientific method is based on the claim and refutation of *evidence* that is collected to support the answers, thus, having obtained an answer, the desirable next goal is to use this experience, which is now part of the knowledge. In the clinical setting, as we have already said, we can use knowledge to prevent evolution toward disease, better identify the causal process of injury and/or illness, and/or to develop/use appropriate care. As we are in an *open thermodynamic system* where life, by using the *individual adaptation pattern*, tries, temporarily, to curb the drift toward chaos (9), any clinical decision, taken in the patient's interest, follows this general *attempt* to gain persistence and maintain order by capitalizing on the previous experience. This issue is a *generalization* of results obtained from population averages to individuals based on the similarity of the clinical profile. Indeed, in clinical epidemiology, it is generally assumed, for example, that “*the risk of a disease at equal levels of known risk factors is similar in any individual belonging to the studied population”* (12), but this is not the truth since individual susceptibility is different from the collective one. Furthermore, the *predictability* of (any) previous clinical experiences is a more complex question since it is based on less/more evidence and weak/strong algorithms applied to set-up an *interpretative model*. It must be said that a model is, always, a subjective interpretation of the researcher, thus a certain bias must be taken into account. Indeed, modeling is subjected to the influence of the *dominant scientific culture* which, inevitably, creates *consensus* on, more or less, conventional interpretative models that are, often, too dogmatic. This is a general question affecting science as a *collective process* that makes it difficult to *falsify the evidence* since consensus is maintained by sociological processes which are explained with organizational and institutional influences (13, 14) (arguments that go beyond this discussion).

In summary, making *forecasts* about the future state of a *thermodynamic system* is at the basis of scientific knowledge, with theoretical and practical implications of the utmost importance that are listed in Table 2A.

**Table 2:** Predicting health and disease.

**A. Why it is difficult to predict the (clinical) future for the individual: the boundary between health and disease is fuzzy.**	
→ Predictability relies on:	
• formulation of relevant questions	
• using appropriate methodology	
• having more evidence	
• strong algorithms	
• interpretative model	
• generalizability of population averages to individuals is problematic:	
→ Interpretative models are:	
• subjective interpretation of the researcher	
• influenced by the dominant scientific culture	
**B. Limitations in the use of statistical models in medicine.**	
• A model is a subjective interpretation of the researcher.	
• A model lacks a complete -structural-systemic- understanding.	
• The predictability of a model relies on algorithms.	
• In statistics, any correlation found does not imply causation.	
• Reproducibility is a standard, mainly, for science.	
• Future exposures may not be predictable precisely.	
• The generalization of results is impossible.	
• The verification of the prevention effectiveness is a complex issue.	
**C. Critical approach to the guidelines system.**	
Multiple instances, difficult to meet at the same time, *built a (better) system of (public) health care*	
• rationalizing the medical intervention	
• reduce costs	
• ensure legal protection of physicians	
• preserve professional autonomy	
• fall within the public/private funding of research	
• ensure an appropriate statistical-mathematical standard	
• check the prevention effectiveness	
**D. Different approaches to the patient if viewed as an individual or as an average.**	
**Individual patient**	**Average patient**
Requires an empathic relationship	Relationship should be limited
Benefit from the consultation of the guidelines	Use of guidelines mandatory
Takes more time	Takes less time
Decision making assumes a high level of responsibility	Responsibilities are shared

### Mathematics at the Bedside and Complexity of Biology

While approaching the complexity of biological life and death, we have no profitable approach except the scientific one that has been applied to the study of biological networks and their relationships. Among the different approaches, *mathematics* is undoubtedly the most profitable in developing *interpretative models* (12) since it has an axiomatic structure, uses logic, and has a method, scientific accuracy, and flexibility.

Indeed, when clinicians use *statistics* to test/develop a model by applying mathematical formulas, they often use a conventional logic that does not fit the *complexity of biology* that goes beyond, but instead follows non-deterministic rules and is characterized by a *non-linear* and, often, *chaotic* dynamics. But this is not a problem for mathematics that is, indeed, flexible; in this regard, one of the applications in biology of a non-linear mathematical model based on attractors of chaos - an attractor is a geometric place to which a dynamic system evolves after a long enough time - is the study of heart rate (15), a biological phenomenon known for its high variability (16). Nonetheless, assuming that the chaotic dynamics are appropriate to describe some human phenomena such as diseases, we could only predict events knowing perfectly the *initial conditions* of the system, and this is not always the case. The lack of knowledge of the initial conditions, obviously, does not rule out that they exist, but we must consider that even at the extreme opposite of *determinism* we can find *non-determinism* or truly *random patterns* that physicists, with the scattering of protons, have suggested to describe the intimate behavior of physical matter (17). Again, is this model suitable for human diseases? The question presents different facets: one is to consider events related to adaptive mechanisms such as *pseudo-random* or phenomena waiting for a new algorithm to formulate a suitable predictive model (11). As humans have a mind oriented to capture causal links and to reject the randomness of natural events, this kind of confidence in the advancement of knowledge allows, temporarily, to avoid the hardness of having *chaos* in the *hospital*. The issue of having a model, however, does not seem to solve the problem as it is, still, a subjective interpretation of the events made by the researcher; thus, *random* or *pseudo-random* are just models. It may be necessary to rethink the science of certainty and that of uncertainty (18) bearing in mind that mathematics is just a tool, it uses logic and creates knowledge by following hypotheses that, primarily, are inspired by *intuition*, a peculiar type of ability that does not use inference or reason (19). Finally, it seems non-trivial to question even the mechanistic approach since it is limited because of the lack of a complete understanding that can result, only, from a broader vision that, till now, seems to have been associated with philosophy. In Table 2B, the main limitations in the use of statistical models in medicine are listed.

### Evidence-Based Guidelines System: From Sources of Bias to Inherent Limitations When Approaching the Individual Patient

The consequences of statistical models at the bedside are *evidence-based guidelines* that are, ideally, useful tools produced to summarize probabilistic data and provide practical guidance. The cultural background which has led to the widespread diffusion of evidence-based guidelines was the setting of the etiological model in favor of a multifactorial one and the progressive adoption of mathematical-statistical models to estimate the risk of an adverse event and to assess possible intervention strategies in order to prevent it (20). This system represents the summation of multiple instances: on the one hand the idea of rationalizing the medical intervention, based on available scientific evidence, in order to contain the costs and to build a better system of health care in the public domain and, on the other hand, to provide a legal protection that allows physicians to preserve a wide professional autonomy. To combine multiple instances is never an easy task, but there are, as we have seen, several drawbacks inherent to the statistical model used in published research since its predictability is, still, limited and, in any case, a research finding provides, only, a partial representation of reality obtained from a *finite* number of subjects. Furthermore, it should also be remembered that in the clinical setting *verification* of the prevention/intervention effectiveness (11) is a complex issue that requires a systematic assessment of its impact on health outcomes with post-study probability testing (21). Another source of bias depends on the fact that scientific research is not a free domain but is subject to public or private funding, according to a complex and questionable interference pattern. Even guidelines, which generally summarize probabilistic data from research studies, are *possibly* biased since they are drafted, mostly, by those who declare a *conflict of interest*; in this regard, several papers are available discussing the issue of financial conflict of interest.

Historically, the need to declare a conflict of interest began in 2003 when the *pharmaceutical industry* established guidelines on *Good Publication Practice* to make the publication of *industry-sponsored trials* more transparent (22). This followed the well-known law suit against *Pfizer* that produced “fraudulent scientific evidence” by “suppressing unfavorable study results to promote off-label uses of gabapentin” (23). This position was followed by the International Committee of Medical Journal Editors requiring, starting from 2004, a registration in a *public trial's registry* as a “condition of consideration for publication” of clinical trials (24). At that time, a review published by *Jama* showed a significant association between industry sponsorship and pro-industry conclusions (25), but the question remains on whether *clinical studies/trials*, even when summarized into practical guidelines, are still now influenced by financial relationship that, “disclosed or undisclosed, relevant or not relevant” have been demonstrated to impact on “whether studies report findings favorable to industry sponsors,” which is supported by a recent survey dated 2019 (26). This potential bias is more evident if we look at the most worldwide prescribed therapies; an 18 year retrospective study supported a significant association between industry-funded *randomized controlled trials* and statistically significant outcomes for antidepressants (27).

Thus, it seems evident that declaring a conflict of interest does not guarantee a lack of bias. Furthermore, a very recent study supported that, years after this obligation, a low percentage of primary studies, such as *randomized controlled trials*, include a declaration of conflict of interest (28). If we look at *research validity*, it can vary considerably; in a meta-analysis of survey data published by PLoS in 2009, “misconducting research” seems to be a fairly common practice that has been *self-admitted*, regardless of the reasons why, by up to 34% of authors explicitly asked about “questionable research practices,” including having *fabricated/falsified* research data or *altered/modified* results to improve the outcome, and 29% of the cases of misconduct known by respondents were never discovered (29). A recent update by the same author of the previous survey supported that *self-admission* rates for both fabrication/falsification, including *plagiarism*, seems to have declined over the years, but *non-self-admission* rates have not changed (30).

In summary, *clinical studies/trials* may not only be *methodologically* incorrect, underpowered, and even misinterpreted (31) but, at the same time, biased by a disclosed or undisclosed conflict of interest and this is, also, for their summarization in *evidence-based guidelines*.

If we assume that available *clinical guidelines* are reliable, as we ideally do expect (32), their widespread diffusion support obvious potential benefits to rationalize medical intervention. Nonetheless, as we stated before, using *heterogeneous information*, made up of more-or-less *valid evidence*, needs a *systematic strategy* for the evaluation/verification of the prevention/intervention effectiveness and the “epistemological responsibility of doctors” when drafting/using guidelines (33).

Finally, a simple question that is scarcely taken into consideration, is that what appears beneficial for our patients *as a group* may not always be suitable for the *individual* and, certainly, not for patients with *comorbidities* (34) and the routine use of guidelines is possibly conflicting with the emerging concept of *personalized medicine* and the model of *shared decision-making* (35).

That is to say, even when using the best on *average* up-to-date evidence, because of the *heterogeneous* response to *any* intervention, we are not able to predict how this strategy may work in a specific patient, even when subgroup analysis is available; thus, it is possible that physicians, informed by last trials' results, but with their direct *clinical experience*, do better than others at prescribing the same evidence-based best option to everyone, failing to profile patients who may not benefit (32). A critical approach to the guidelines system is reported in Table 2C.

## Conclusions as a Starting Point

The complexity of life and death is demonstrated by their unpredictability, thus making predictions on the *clinical future* of an individual seem an arduous task. The individual adaptation profile to environmental changes seems to have part of the answer, but predictability uses statistical modeling that, to increase the rate of probability, needs more subjects, thus moving far away from the individual subject in favor of the collective. This means that the individual subject/patient is, still, missing from research papers and guidelines, appearing only in case reports. This does not mean that forecasts in medicine are banned but, simply, that they have to be brought back to the main watercourse of science that should be understood as a continuous process and, even if mathematics at the bedside could be extremely profitable in developing interpretative models, these models are still subjective, fallible, and, therefore, confutable. Before the advent of *precision medicine*, doctors pursued a relationship with the individual patient, who cannot be reduced to a mathematical average, and to recollect Osler's thoughts when he wrote in his most famous essay, Aequanimitas, delivered to new doctors in 1889 at Pennsylvania School of Medicine: “The practice of medicine is an art, based on science.” That is to say, medicine is not an art like painting but, neither is it a science like physics; it needs humanity, empathy, respect, communication, and *fact checking ability* when using evidence- based medicine algorithms for the individual patient to plan a strategy and reach the best outcome.

In Table 2D the different approaches to the patient if viewed as an individual or as an average is summarized.

## Limitation of the study

Perspectives are intrinsically limited but, sometimes, useful.

## Data Availability Statement

Publicly available datasets were analyzed in this study. All datasets generated for this study are included in the article/supplementary materials.

## Author Contributions

The author confirms being the sole contributor of this work and has approved it for publication.

## Conflict of Interest

The author declares that the research was conducted in the absence of any commercial or financial relationships that could be construed as a potential conflict of interest.
